# Role of Pharmacogenetics in Hematopoietic Stem Cell Transplantation Outcome in Children

**DOI:** 10.3390/ijms160818601

**Published:** 2015-08-10

**Authors:** Raffaella Franca, Gabriele Stocco, Diego Favretto, Nagua Giurici, Giuliana Decorti, Marco Rabusin

**Affiliations:** 1 Institute for Maternal and Child Health (I.R.C.C.S.) Burlo Garofolo, UO Pediatric Hemato-Oncology, Trieste 34137, Italy; E-Mails: dieg.o@libero.it (D.F.); nagua.giurici@burlo.trieste.it (N.G.); marco.rabusin@burlo.trieste.it (M.R.); 2 Department of Life Sciences, University of Trieste, Trieste 34127, Italy; E-Mails: stoccog@units.it (G.S.); decorti@units.it (G.D.)

**Keywords:** hematopoietic stem cell transplantation, pharmacogenetics, graft *vs.* host disease, sinusoidal obstructive syndrome

## Abstract

Hematopoietic stem cell transplantation (HSCT) is an established therapeutic procedure for several congenital and acquired disorders, both malignant and nonmalignant. Despite the great improvements in HSCT clinical practices over the last few decades, complications, such as graft *vs.* host disease (GVHD) and sinusoidal obstructive syndrome (SOS), are still largely unpredictable and remain the major causes of morbidity and mortality. Both donor and patient genetic background might influence the success of bone marrow transplantation and could at least partially explain the inter-individual variability in HSCT outcome. This review summarizes some of the recent studies on candidate gene polymorphisms in HSCT, with particular reference to pediatric cohorts. The interest is especially focused on pharmacogenetic variants affecting myeloablative and immunosuppressive drugs, although genetic traits involved in SOS susceptibility and transplant-related mortality are also reviewed.

## 1. Introduction

Hematopoietic stem cell transplantation (HSCT) is an established therapeutic procedure that consists of the intravenous infusion of autologous or allogeneic hematopoietic progenitor cells to reinstate the normal hematopoietic functions in patients whose bone marrow is compromised. Several congenital and acquired disorders, both malignant and nonmalignant, are treated with HSCT, and the frequency of transplants is increasing worldwide from year to year: according to the European Society of Blood and Marrow Transplantation (EBMT) reporting on the annual activity in 48 European (or affiliated) countries, 37,818 HSCTs were carried out in 33,678 adult patients in 2012, with a 6% increase in comparison to 2011. Less than half (14,165, 42%) received allogenic HSCT. In the same year, 4041 children under 18 years of age underwent their first HSCT: most of these pediatric transplants were allogeneic HSCT (2877, 71%) and had been indicated for children affected by immunological nonmalignant conditions and hematological diseases rarely occurring in the adult population (acute lymphoblastic leukemia (ALL, ~26%), primary immune deficiencies (~16%), acute myeloid leukemia (AML, ~14%) and bone marrow failure (~12%), as reported for allogeneic HSCT performed in 109 dedicated European pediatric transplant centers). The remaining 1164 pediatric patients (29%) underwent autologous HSCT, mainly because of solid tumors (~66%) and lymphomas (~15%) [1]. In both pediatric and adult allogeneic transplants, half of the donors were family related; as a stem cell source, bone marrow was indicated for children (~63%), whereas peripheral blood was mainly employed for adults (~72%).

In the field of allogeneic HSCT, complications are common: infections, development of acute and chronic graft *vs.* host disease (aGVHD and cGVHD) and organ failure, such as sinusoidal obstructive syndrome (SOS), represent the major causes of morbidity and mortality [2]. The onset of these complications depends on several clinical and biological factors, including the type and stage of disease, comorbidities, age, sex mismatching, conditioning regimes, cytomegalovirus status and stem cell source. The individual genetic background could also influence the success of transplantation. In particular, pharmacogenetic variants in genes encoding for drug metabolizing enzymes and transporters might contribute to different pharmacokinetic profiles and might affect drug effects and/or safety. Prior to HSCT, aggressive conditioning regimens are required for destroying patient bone marrow to eradicate underlying disease, for immunosuppressing the host to prevent rejection and for creating a stem cell niche to allow engraftment of donor cells. Traditionally, conditioning includes high-dose (8–10 Gy) total body irradiation (TBI) or myeloablative chemotherapy with busulfan (BU) and cyclophosphamide (CPM). Recently, reduced-intensity conditioning based on low-dose TBI (2–3 Gy), fludarabine eventually combined with chemotherapeutic drugs (such as BU, cytarabine and idarubicin) and treosulfan have been introduced: their use has resulted in a higher frequency of graft failure; therefore, these regimens require further optimization [3]. After HSCT, immunosuppressive drugs, such as calcineurin inhibitors (cyclosporin A (CsA) or tacrolimus (Tac)), glucocorticoids (GC) and methotrexate (MTX), are administered to prevent GVHD. Myeloablative and immunosuppressant agents have low therapeutic indexes, and their plasma concentrations are associated with patient clinical course: subtherapeutic levels have been associated with an increased risk of graft failure (acute rejection and relapse), while overdosing has been associated with a greater incidence of transplant-related toxicities (infections, GVHD and SOS) [4]. In order to achieve and/or maintain the optimal therapeutic range of myeloablative and immunosuppressive drugs, doses are adjusted on the basis of pharmacokinetic parameters [4]. However, the therapeutic drug monitoring (TDM) does not prevent the drug-related adverse effects, in particular those occurring after the first administration, suggesting the need of an *a priori* prediction of patient response (for example, by pharmacogenetic markers) in order to define the right initial dose. Moreover, pharmacogenetic markers associated with treatment outcome could be useful to stratify patients to achieve earlier the maximal therapeutic benefit.

Nowadays, some pharmacogenetic test are integrated into routine clinical care: the best characterized example is thiopurine methyl transferase (TPMT), whose polymorphisms affect mercaptopurine metabolism and for which genotype-based dosing clinical guidelines are now available [5]. Several studies, mainly based on candidate gene/pathway approaches, have been performed to investigate the role of genetic variants, in particular single-nucleotide polymorphisms (SNPs), in HSCT outcome; however, relatively few associations have been found, and none have been consistently validated so far. Therefore, no pharmacogenetic test is currently integrated in HSCT protocols [6].

In the allogeneic HSCT setting, the genetic contribution goes beyond the inter-patient variability in response to myeloablative and immunosuppressive treatments. Donor genetic background should also be taken into account, and particularly, the genetic discrepancies between donor and recipient are able to strongly influence the final outcome. Indeed, patients routinely receive HSCT from human leukocyte antigens (HLA) genotypically-identical sibling or from a HLA phenotypically-matched unrelated donors, to lower the risk of graft rejection and GVHD. In patients with leukemia receiving myeloablative conditioning, the rejection rate is 0.1% in patients given HLA identical sibling transplants, compared to 5% in those given HLA-mismatched grafts [7,8]. HLA genes encode cell-surface proteins responsible for antigen peptide presentation in adaptive immune response. The HLA genes are the best characterized genes in the major histocompatibility complex (MHC). Non-HLA genes are currently not considered in donor selection; however, polymorphic discrepancies between donor and recipient in these genes might result in allopeptides still able to trigger an adverse immune reaction in patients after HSCT. These minor histocompatibility antigens (miHA) are the result of either non-synonymous SNPs (encoding for a protein with a single amino acid difference), or base-pair insertion-deletion (Ins/Del) variants in coding exons (resulting in a frame-shift reading), or copy number variation (where the protein is missing in individuals with homozygous deletion of the gene) in the genomes of the donor and recipient [9]. miHA-mediated alloimmunity has been thus correlated mainly with the development of severe GVHD, as already reported in the literature [10].

The purpose of this review is to summarize the recent available literature on candidate genes influencing HSCT outcome and on the predicting role of their polymorphisms in GVHD, SOS and transplant-related mortality (TRM). Our interest is especially focused on pediatric HSCT cohorts and pharmacogenetic variants, although limited literature is available. Particular attention will be given to drugs and regimens currently used in the protocols proposed by the Associazione Italiana di Ematologia e Oncologia Pediatrica (AIEOP).

## 2. Graft *vs.* Host Disease

Despite major improvements in clinical practice over the past few decades, clinical GVHD remains a complex post-transplant complication, even in HLA-matched allogeneic HSCT. Severe aGVHD occurs within 100 days in 11%–50% of pediatric HSCT patients, while cGVHD occurs after 100 days with an incidence of 5%–60% [11,12]. GVHD arises in the immune-compromised host when newly-engrafted competent donor T cells undergo clonal expansion in response to recipient alloantigens; this immuno-activation ends up in T cells’ migration to, and damage of, host normal lymphoid organs and target tissues (particularly skin, liver and gastrointestinal tract).

### 2.1. Treatment Protocols

Guidelines for GVHD prevention and treatment have been recently proposed by EBMT and European Leukemia Net (ELN) [13]. These recommendations have been developed for common standard risk allogeneic HSCT in adults, whereas EBMT-ELN guidelines for pediatric transplantations are still not available. In Italy, HSCT on pediatric patients has been carried out according to AIEOP common guidelines since 1986. Although preparative and prophylactic AIEOP-HSCT protocols depend on patient underlying diseases, age and institutional choice, they are substantially homogeneous among Italian centers. Between 1990 and 1997, GVHD prophylaxis consisted mostly of CsA alone (1–3 mg/kg i.v.) for patients undergoing allogeneic HSCT from an HLA compatible-related donor, and in CsA at higher doses (2–3 mg/kg i.v.) plus short-course MTX in patients transplanted from unrelated donors, as reported for a cohort of 420 children affected by acute leukemia and enrolled in 13 different AIEOP hospitals [14]. In recent years, anti-thymocyte globulin (2.5–3.75 mg/kg/day from Day −4–Day −2) has been introduced and is used in combination with CsA and MTX for the majority of allogeneic HSCT from an unrelated donor, as reported in three different cohorts of ALL patients (211 high-risk in first complete remission, 69 Philadelphia-positive and 395 in second complete remission enrolled in 19, 11 and 18 different AIEOP Centers, respectively) transplanted between 1990 and 2008 [15,16,17].

### 2.2. Variants Affecting Graft vs. Host Disease

A PubMed search performed in January 2015 using the term “GVHD and polymorphism and hematopoietic stem cell transplantation” returned 67 entries in the last five years. A wide range of variants throughout several different genes has been considered across studies, with few overlaps in candidate polymorphisms.

Genes and variants investigated because of their role in the immune response and in inflammation are beyond the purpose of this review. Interestingly, a number of studies have been published, characterizing donor and recipient HLA allelic mismatch at the single nucleotide level for the discovery of clinically-important human genetic variation with a role in aGVHD development. In a discovery-validation study, 4205 patients affected by blood malignancies and their unrelated donor matched for five classical *HLA* genes (*HLA-A*, *-B*, *-C*, *-DRB1* and *-DQB1*) were genotyped for 1220 MHC region SNPs using a specific Illumina^®^ platform [18]. Pair mismatch in rs2281389 (2 kb centromeric to *HLA-DPB1*) conferred an increased risk of Grades II–IV aGVHD [18], independently of the *HLA-DPB1* mismatch that represents a risk factor for aGVHD *per se* [19]. The same authors retrospectively assessed 1108 MHC SNPs (1078 in common with the previous study) in another HSCT cohort of 2628 patients and unrelated donors mismatched at one of the classical HLA genes. The previously identified rs2281389 showed only a suggestive effect for aGVHD [20]. In the multi-SNP analysis, 12 SNPs were associated with transplant outcome after Bonferroni correction. Concerning Grades III–IV aGVHD, rs209130 pair mismatch, rs2075800 patient genotype and rs394657 donor genotype emerged as significant genetic markers. Other reports focused on non-classical MHC (class Ib) molecules, such as *HLA-E* and *HLA-G* genes. In contrast to class Ia antigens, class Ib molecules seem less important for transplant rejection, since they are highly conserved and exhibit limited protein variability. So far, no association has been found between *HLA-E* gene polymorphisms and transplant outcome [21,22], whereas controversial results were reported for a 14-base pair (bp) Ins/Del polymorphism located at the 3′ untranslated region of exon 8 in the *HLA-G* gene. HLA-G is expressed in several membrane-bound or soluble isoforms, these latter displaying an immunosuppressive role so that higher plasma levels correlate with reduced transplant rejection: the 14 bp Ins allele has been associated with a decreased mRNA stability and thus to lower protein expression [23,24]. Indeed, in the study of Boukouaci *et al.*, analyzing the distribution of this variant in a HSCT population of 157 sibling donor/recipient pairs fully matched for *HLA-G* genotypes, the homozygous state for the 14-bp Ins allele resulted in a risk factor for Grades III–IV aGvHD in multivariate analysis [25]. Caocci *et al.* found that the homozygosity for the 14-bp Del in HSC-unrelated donors was independently and significantly correlated to aGVHD in a HSCT cohort of 78 β-thalassemia major patients, whereas Chiusolo *et al.* did not find any correlation between this polymorphism and the risk of GVHD [26,27]. Although no genetic trait arising from these studies has reached clinical significance yet, further pharmacogenetic investigation is required in this field. Indeed, *HLA* and SNP mismatches conferred independent risks of GVHD, thus the identification of unfavorable or favorable polymorphic genotypes in *HLA* genes might help with estimating risk prior to transplantation or might help with selecting the most proper donors, even in serologically HLA-matched pairs.

This review focuses on genes and variants affecting drug pharmacokinetic parameters and pharmacodynamic properties of immunosuppressants or myeloablative regimens in addition to clinical phenotypes. The articles considered are summarized in Table 1.

**Table 1:** Genetic association studies with graft *vs.* host disease (GVHD) and pharmacokinetic parameters in hematopoietic stem cell transplantation (HSCT) studies.

Gene	Variant	Sample Size	Age	Ethnicity	GVHD	Genotype Effect on PK Parameters	Reference
*ABCB1*	rs1128503 (C1236T)	58 R	A	J	None	None on CsA or Tac initial concentration	[28]
	rs2032582 (G2677TA)	58 R	A	J	None	None on CsA or Tac initial concentration	[28]
		20 R	A	K	–	None on MTX CL	[29]
		27 R	P	J	–	None on Tac CL	[30]
	rs3213619 (T-129C)	27 R	P	J	–	None on Tac CL	[30]
	rs1045642 (C3435T)	27 R	P	J	–	None on Tac CL	[30]
		20 R	A	K	–	CC/CT ↑ MTX CL	[29]
		58 R	A	J	None	None on CsA or Tac initial concentration	[28]
*ABCC2*	rs717620 (C-24CT)	20 R	A	K	–	None on MTX CL	[29]
	rs2273697 (G1249A)	20 R	A	K	–	None on MTX CL	[29]
*ABCG2*	rs2231142 (C421A)	27 R	P	J	–	None on Tac CL	[30]
*ATIC*	rs2372536 (C347G)	20 R	A	K	–	None on MTX CL	[29]
*CYP2C19*	rs4244285 (G681A, *2)	58 R	A	J	None	None on CsA or Tac initial concentration	[28]
*CYP3A5*	rs15524 (A>G)	58 R	A	J	None	TT ↑ CsA initial concentration	[28]
	rs4646450 (G>A)	58 R	A	J	None	CC ↑ Tac initial concentration and ↓ Tac dosage from Day −1–Day +28	[28]
	rs3800959 (A>G)	58 R	A	J	None	none on CsA or Tac	[28]
	rs776746 (A6986G)	58 R	A	J	None	GG ↑ CsA and Tac initial concentration; GG ↓ Tac dosage from Day −1–Day +28	[28]
		27 R	P	J	None	None on Tac CL	[30]
*GGH*	rs3758149 (C-401T)	20 R	A	K	–	None on MTX CL	[29]
*GST-A1*	rs3957356 (G-52A)	18 R †	P	Ar	None	*B*B ↓ oral BU Cmax, AUC and CL/kg body weight	[31]
	rs3957357 (C-69T, *B)	69 R	P	C	None	*B none on BU-PK	[32]
*GST-M1*	Deletion	18 R †	P	Ar	None	none on BU-PK	[31]
		69 R	P	C	Null ↑		[32]
*GST-T1*	Deletion	18 R †	P	Ar	None	None on BU-PK	[31]
*GST-P1*	rs1695 (A313G)	18 R †	P	Ar	None	Oral BU-Cmax and AUC	[31]
*MTHFR*	rs1801133 (C677T)	20 R	A	K	–	None on MTX CL	[29]
	rs1801131 (A1298C)	20 R	A	K	–	None on MTX CL	[29]
*TYMS*	rs34743033 (28 bp tandem repeat)	20 R	A	K	–	None on MTX CL	[29]

A, adults (≥18 years); *ABCB1* (MDR1), ATP-binding cassette, sub-family B, member 1 (multi drug resistance protein); *ABCC2*, ATP-binding cassette, sub-family C, member 2; *ABCG2*, ATP-binding cassette, sub-family G, member 2; AL, acute leukemia; Ar, Arabs; *ATIC*, 5-aminoimidazole-4-carboxamide ribonucleotide formyltransferase/IMP cyclohydrolase; AUC, area under the curve; BU, busulfan; C, Caucasians; CL, clearance; Cmax, maximal concentration; CsA, cyclosporin-A; *CYP2C19*, cytochrome P450, family 2, subfamily C, member 19; *CYP3A5*, cytochrome P450, family 3, subfamily A, member 5; D, donor; *GGH*, gamma-glutamyl hydrolase; *GST*, glutathione-*S*-transferase; J, Japanese; K, Korean; *MTHFR*, methylenetetrahydrofolate reductase; P, pediatric (<18 years); MTX, methotrexate; PK, pharmacokinetics; R, recipient; *Tac*, tacrolimus; *TYMS*, thymidine phosphorylase; †, patients affected by congenital hemoglobinopathy; ↑, increase; ↓, decrease.

Onizuka *et al.* analyzed 58 serial cases of adult Japanese patients who underwent HSCT between 2006 and 2009: thirty-seven (64%) received Tac (starting dose of 0.02 mg/kg by continuous i.v. infusion from Day −1) and a short course of MTX (15 mg/day i.v. on Day +1 and 10 mg/day i.v. on Days +3 and +6) as GVHD prophylaxis; the other group (21 cases, 36%) received CsA (starting dose of 1.5 mg/kg, twice daily by 3 h i.v. infusion from Day −1) [28]. A broad overview of the pharmacological profiles of calcineurin inhibitors in transplant patients has been recently published by the Pharmacogenomics Knowledge Base (PharmGKB) [33]. Variability in drug disposition has been attributed to interindividual differences in the expression of the metabolizing enzymes cytochrome P450 (CYP, particularly the 3A4 isoform for CsA and the 3A5 isoform for Tac) and in the expression of the drug transporter P-glycoprotein (encoded by the *ABCB1* gene, formerly known as the multidrug resistance 1 gene (*MDR1*)). Onizuka *et al.* found that initial plasma concentration of calcineurin inhibitors was significantly affected by *CYP3A5*, but not *ABCB1*, polymorphisms: the CsA concentration measured at Day +2 or Day +3 was significantly increased in mutated rs776746 GG carriers (250.8 ± 69.9 *vs.* 138.6 ± 30.8 ng/mL in GA/AA, *p* = 0.027) and rs15524 TT genotype (251.1 ± 92.8 *vs.* 145.7 ± 36.7 ng/mL in TC/CC, *p* = 0.044; C>T SNP in the 3′UTR of the gene). The rs776746 is an A to G change at position 6986G in intron 3, particularly frequent in Caucasians, that results in an altered RNA splicing site and in a nonfunctional protein: subjects with the GG genotype (CYP3A5*3/*3) are considered CYP3A5 non-expressors, in contrast to those carrying at least one A allele (CYP3A5*1) [34]. The SNP rs15524 is a T to C common variant located in the 3′ untranslated region. For Tac, the initial serum concentration was also significantly increased in mutated rs776746 GG subjects (19.7 ± 2.4 *vs.* 13.4 ± 3.1 ng/mL in AG/AA, *p* = 0.013) and in CC carriers of the intronic rs4646450 (20.3 ± 2.5 *vs.* 13.8 ± 2.7 ng/mL in CT/TT, *p* = 0.0058, T>C SNP). TDM was performed three times a week, adjusting dosage by 20% in order to maintain the therapeutic target concentration of 250–300 ng/mL for CsA and 10–15 ng/mL for Tac. The pharmacogenetic contribution to drug adjustment, measured as the Day +28/Day −1 drug dose ratio, revealed that Tac was remarkably reduced in *CYP3A5* rs4646450 CC (39.8% ± 8.4% *vs.* 86.1% ± 25.5% for TC/TT, *p* = 0.001) and rs776746 GG patients (41.6% ± 8.2% *vs.* 90.5% ± 29.4% for AG/AA, *p* = 0.0021), consistent with the role of these genotypes in compromising the CYP3A5 metabolic capability and with the role of CYP3A5 on Tac metabolism [28]. *CYP3A5* rs776746 was also investigated in a small population of 27 pediatric Japanese patients with blood disorders transplanted between 2003 and 2009 who underwent a similar GVHD prophylaxis: in contrast to what was observed in adults, Tac dose, blood concentration and clearance (CL) were not significantly affected by this SNP, comparing children with GG and AA/AG genotypes [30]. These results suggest the important issue of age as a confounding variable in pharmacokinetic analysis. Indeed, beside the presence of poor or null metabolizing variants in *CYP3A* genes, the CYP3A isoforms themselves are not uniformly expressed from the prenatal period to adulthood and show ontogenetic variation [35]. *ABCB1* rs1128503, rs2032582 and rs3213619 were not affecting Tac pharmacokinetics, neither in adults nor in children [28,30]. Another study investigated the MTX pharmacogenetic/pharmacokinetic profiles in a small population of 20 Korean adults receiving HSCT between 2009 and 2011, mainly because of malignant diseases [29]. Patients were treated with CsA (5 mg/kg/day from Day −1–Day +3 and then 3 mg/kg/day up to 14 days post-HSCT, CsA dosage adjusted to maintain a 150–300 ng/mL blood concentration) and intravenous MTX (at 15 mg/m^2^ on Day +1 and at 10 mg/m^2^ on Days +3, +6 and +11). A marked inter-individual variability was shown after the first MTX administration (560–2089 ng/mL in measurements at five minutes from the beginning of the infusion). In a covariate adjusted model, mean MTX CL was 7.08 L/h with a 21.6% inter-individual variability: this parameter showed a significant increase of ~32% in patients with a 10 mL/min increase in the glomerular filtration rate and a significant decrease of ~61% in those with the concomitant administration of penicillin. Patients were genotyped for five common SNPs in four genes of the folate metabolic pathway (*ATIC* rs2372536, *MTHFR* rs1801131 and rs1801133, *GGH* rs3758149 and *TYMS* rs34743033) and four SNPs in two MTX transporters (*ABCB1* rs2032582 and rs1045642, *ABCC2* rs717620 and rs2273697). Mean MTX CL was significantly increased of ~21% in patients with the *ABCB1* rs1045642 CC/CT genotypes compared to those with the TT genotype (10.07 and 8.26 L/h; *p* < 0.001). SNP rs1045642 (3435C>T) is well-studied and was shown to reduce intestinal P-glycoprotein expression and function and, therefore, had the potential to affect drug bioavailability [36].

Elashid and coworkers retrospectively investigated 18 Arab children, HSC transplanted for congenital hemoglobinopathies between the years 2002–2008. All of these patients were treated with a myeloablative conditioning regimen based on high-dose oral BU (1 mg/kg actual body weight every 6 h for 4 days), followed by intravenous cyclophosphamide (CPM), and were genotyped for the most common polymorphisms in phase II detoxifying enzymes glutathione-*S*-transferases (GSTs). Particularly, SNP rs3957356 (−52 C>T, GSTA1*B) in the *GSTA1*, SNP rs1695 (A*>*G, 105Ile > Val) in the *GSTP1* and deletions in both the *GSTM1* and *GSTT1* genes were characterized [31]. This study is limited by the small number of patients enrolled and the weakness of statistical analysis. However, all of the patients who developed GVHD (*n* = 5, 27.7%), both acute and/or chronic, had in common the *GSTM1*-null genotype; four were *GSTT1*-positive. In a pharmacogenetic analysis on *GST* polymorphisms and HSCT outcome in a pediatric cohort of 69 HSCT patients, Ansari and coworkers found in a multivariate analysis that *GSTM1*-null subjects developed more GVHD (*p* = 0.03). However, this result was significant only for children older than four years [32].

## 3. Sinusoidal Obstruction Syndrome

Severe SOS, also known as veno-occlusive disease, is among the most serious complications of HSCT, representing a common cause of transplant-related death, with reported mortality rates of up to 50% and more. The incidence rate ranges from 5%–41% of transplanted patients, depending on the characteristics of the patients enrolled, the type of transplant, the conditioning regimens and the diagnostic criteria used [37]. Clinically, SOS occurs within 3–4 weeks after HSCT and is characterized by painful hepatomegaly, jaundice, ascites and fluid retention; the most severe episodes are life threatening conditions leading to death for progressive multi-organ failure [38]. The pathogenic mechanism of SOS is not fully understood yet; however, it is known that it is the result of a progressive damage in sinusoidal endothelial cells and hepatocytes, caused by an out-of-control inflammatory process initiated at the centrilobular zone in the hepatic acinus. The epithelium’s injury is accompanied by the secretion of inflammatory cytokines and vasoactive mediators, subendothelial deposition of clotting factors (*i.e*., large von Willebrand factor multimers, factor VIII and fibrin) and activation of the coagulation cascade, which leads to a progressive sinusoidal obstruction. Risk factors for SOS are related to both pre-transplant medical history (e.g., pre-existing disease and/or liver damage, younger age in pediatric patients, high serum ferritin) and transplant process (e.g., conditioning therapy, pre-transplant medications) [39]. Diagnosis of SOS relies on clinical criteria that are generally either the modified Seattle or the Baltimore ones [40,41,42]. Both diagnostic systems are based on the occurrence of jaundice, weight gain, hepatomegaly and ascites within the first three weeks after HSCT. As recently highlighted by EBMT, these criteria are limited in sensitivity and specificity; being developed more than 20 years ago, they are outdated and need to be rethought on the basis of the current HSCT procedures that now include alternative donors, reduced intensity regimens and new drugs for SOS handling [43]. Other conditions, such as aGVHD, viral and fungal infections, drug-induced cholestatic hepatitis (fluconazole, itraconazole, trimethoprim) and Budd-Chiari syndrome represent SOS confounders [44]. So far, veno-occlusive disease has no standardized prophylaxis or treatment in HSCT settings. The most promising agent is defibrotide, which was approved in 2014 by the European Medicines Agency for the treatment of severe SOS and is now being investigated for its role in endothelial syndrome prevention [43].

Although this review focuses mainly on pharmacogenetic variants able to influence SOS, a section dedicated to polymorphisms associated with SOS susceptibility will also be reported below. Studies of interest are summarized in Table 2.

**Table 2:** Genetic association studies with sinusoidal obstructive syndrome (SOS) in HSCT.

Gene	Variant	Sample Size	Age	Ethnicity	SOS	Reference
*ACE*	Intron 16 I/D	89 D/R	P/A	C	None	[45]
*CPS*	rs1047891 (Thr1405Asn)	168 R	A	nk	**CPS 1405Asn allele + HFE 282Tyr allele ↓**	[46]
*CYP2B6*	*2 (C64T, Arg22Cys)	107 D/R leuk	P/A	nk	None	[47]
	*3 (C777A, Ser259Arg)	107 D/R leuk	P/A	nk	None	[47]
	*4 (A785G, Lys262Arg)	107 D/R leuk	P/A	nk	None	[47]
	*5 (C1459T, Arg487Cys)	107 D/R leuk	P/A	nk	None	[47]
		66 R	P	C	None	[48]
	*6 (G516T, Gln172His)	107 D/R leuk	P/A	nk	**GG genotype in both D and R ↑**	[47]
	*9	66 R	P	C	NONE	[48]
*CY2C19*	*2	66 R	P	C	NONE	[48]
	*17	66 R	P	C	NONE	[48]
*CY2C9*	*2	66 R	P	C	None	[48]
	*3	66 R	P	C	None	[48]
*F2*	rs1799963 (G20210A)	89 D/R	P/A	C	None	[45]
		209 R	A	C	(A allele ↑)	[49]
		10 R	P	nk	None	[50]
*F5*	rs6025 (G1691A)	89 D/R	P/A	C	None	[45]
		209	A	C	None	[49]
		10	P	nk	(A allele ↑)	[50]
*FGB*	rs1800790	89 D/R	P/A	C	None	[45]
*FMO*	rs2266780	66 R	P	C	None	[48]
	rs2266782	66 R	P	C	None	[48]
	rs1736557	66 R	P	C	None	[48]
*GPIIIa*	PIa1/a2	89 D/R	P/A	C	None	[45]
*GSTA1*	rs3957356 (−52 C>T)	18 R*	P	A–M	None	[31]
		69 R	P	C	None	[32]
	rs3957357 (C-69T,*B)	77 R	P	Mixed	None	[51]
		69 R	P	C	**TT↑**	[32]
	rs11964968 (T-513C)	69 R	P	C	None	[32]
	rs4715332 (T-567G)	69 R	P	C	None	[32]
	rs4715333 (T-631G)	69 R	P	C	None	[32]
	rs58912740 (C-1142G)	69 R	P	C	None	[32]
*GSTM1*	Deletion	69 R	P	C	None	[32]
	Deletion	18 R*	P	A–M	None	[31]
	Deletion	77 R	P	Mixed	None (↑ univariate)	[51]
	Deletion	114 R	P	Indian	**Null↑**	[52]
	Deletion	107 D/R leuk	P/A	nk	None	[47]
*GSTP1*	rs1695 (A313G, Ile105Val)	69 R	P	C	None	[32]
		18 R*	P	A–M	None	[31]
		77 R	P	Mixed	None	[51]
		107 D/R leuk	P/A	nk	None	[47]
	rs1138272	69 R	P	C	None	[32]
*GSTT1*	Deletion	18 R*	P	A–M	None	[31]
		77 R	P	Mixed	None	[51]
		114 R	P	Indian	None	[52]
		107 D/R leuk	P/A	nk	None	[47]
*HFE*	rs1800562 (Cys282Tyr)	168 R	A	nk	**282Tyr allele ↑**	[46]
*HSPE*	rs4693608 (G>A)	160 R	P	C	**G allele ↓**	[53]
	rs4364254 (C>T)	160 R	P	C	**C allele ↓**	[53]
*IL-1β*	rs16944 (C-511T)	76 D/R	P	nk	**TT genotype in donor ↑**	[54]
*MTHFR*	rs1801133 (677C>T)	107 D/R leuk	P/A	nk	None	[47]
	rs1801133 (C677T)	89 D/R	P/A	C	None	[45]
	rs1801131 (1298A>C)	107 D/R leuk	P/A	nk	None	[47]
*PAI-1*	rs1799889	89 D/R	P/A	C	None	[45]
*VDR*	rs1544410 (BsmI G>A)	107 D/R leuk	P/A	nk	None	[47]
	rs7975232 (ApaI G>T)	107 D/R leuk	P/A	nk	None	[47]
	rs731236 (TaqI T>C)	107 D/R leuk	P/A	nk	None	[47]

A, adults (≥18 years); *ACE*, angiotensin converting enzyme; A–M, Arab Muslim; C, Caucasians; D, donor; P, pediatric (<18 years); R, recipient; *CPS*, carbamoyl-phosphate synthetase; *CYP*, cytochrome P450; *F2*, prothrombin; *F5*, factor V (Leiden); *FGB*, fibrinogen-β-chain; *FMO*, flavin-containing monooxygenase; *GPIIIa*, glycoprotein IIIa; *GST*, glutathione-*S*-transferase; *HFE*, hemochromatosis; *HSPE*, heparanase; *IL*, interleukin; leuk, leukemic patients; *MTHFR*, methylene tetrahydrofolate reductase; *PAI-1*, plasminogen activator inhibitor 1; *VDR*, vitamin D receptor; * patients affected by congenital hemoglobinopathy; nk, not known; Texts written in bold type refers to significant findings.

### 3.1. Pharmacogenetics of SOS

High intensity conditioning regimens, such as high dose total body irradiation and myeloablative regimens, including BU and CPM, have been correlated to SOS incidence. Furthermore, medications, such as chemo- and radio-therapeutics or antivirals used in treatments prior to transplantation in patients affected by malignancies, are well-known agents triggering SOS [55].

#### 3.1.1. Busulfan

Busulfan, whose chemical designation is 1,4-butanediol dimethanesulfonate, is a bifunctional alkylating agent with a high biological activity in HSCT. BU cytotoxicity is likely mediated by the formation of DNA cross-links, mainly intra-strand ones, involving guanine N7 [56]. BU plasma concentrations correlate with HSCT clinical outcomes: when BU is administered as conventionally scheduled in myeloablative protocols (16 doses over four days), steady state concentration should fall in a narrow therapeutic window between 615 and 1026 ng/mL in order to achieve a selective and effective suppression of bone marrow. There is significant inter-patient variability in BU pharmacokinetics. BU steady-state levels lower than 600 ng/mL might result in graft rejection and relapse, whereas BU levels exceeding the upper therapeutic limit of 900 ng/mL lead to an increased SOS incidence and GVHD toxicity. An excellent review summarizing BU pharmacogenetic/pharmacokinetic studies and HSCT outcome, with particular reference to pediatric cohorts, has been recently published [4].

BU is inactivated primarily in the liver through the formation of a BU sulfonium ion conjugate in a reaction catalyzed by GST, mainly the GST-A1 isoform (the most abundant GST expressed in the liver) and, to a lesser extent, GST-P1, GST-M1 and GST-T1. Ansari *et* *al.* investigated the relationship between SNPs in *GSTA1* (rs3957357, −69C>T tagging SNP of the haploblock with rs3957356, −52G>A and rs4715332, −567T>G; rs11964968, −513T>C; rs4715333, −631T>G; rs58912740, −1142C>G) and in *GST-P1* (rs1695, 313A>G and rs1138272, 341C>T), as well as the homozygous deletion of *GSTM1* (null genotype) with first-dose BU pharmacokinetic parameters and outcome in 69 children undergoing HSCT between May 2000 and August 2010. Myeloablative conditioning consisted of 2 h BU-infusion every 6 h from Day −9–Day −6, followed by i.v. CPM. Prophylaxis for GVHD (CsA plus MTX or steroids), seizures (lorazepam, midazolam), infections (fluconazole, acyclovir, trimethoprim/sulfamethoxazole) and SOS (ursodeoxycholic acid) was given [32]. SOS was diagnosed according to the Seattle criteria. Patients carrying the −69TT genotype (identifying the GSTA1*B haplotypes with a 40%–42% frequency in Caucasians) had five-fold higher occurrence of SOS in comparison to others (Hazard ratio (HR) = 5.3; 95% Confidence Interval (CI) = 1.3–21.5, *p* = 0.009) with a more evident effect in girls. Hepatic GST-A1 protein expression is decreased significantly in GSTA1*B subjects [57], suggesting that the higher SOS incidence could be due to higher BU levels in these individuals. However, no correlation was found between GSTA1*B and BU pharmacokinetic parameters. In contrast, lower drug levels and higher CL (*p* ≤ 0.03) were observed in individuals homozygous for GSTA1*A allele subtype 2 (−69C, −613G), who showed also better event-free survival (*p* = 0.03) in comparison to patients without the indicated genotype. *GSTA1* polymorphisms were characterized in other pediatric cohorts: most of these studies agreed on the lower BU CL of the GSTA1*B individuals, but did not find an association with SOS (as reviewed in [4]). Ansari and coworkers found also that *GSTM1*-null patients older than four years had higher BU levels and lower CL (*p* ≤ 0.04) [32]. Similarly, in 18 Arab children who underwent allogeneic HSCT due to congenital hemoglobinopathy, *GSTM1*-null individuals showed lower BU-AUC/Kg compared to *GST-M1*-positive individuals [31]. In a pharmacokinetic-pharmacogenetic model developed for a cohort of 77 children and young adults, white and non-white patients diagnosed with malignant hematologic or nonmalignant diseases, neither the deletion of *GSTM1* nor the other *GST* polymorphisms seemed to correlate with BU pharmacokinetics as important covariates. Only in the univariate analysis, *GSTM1*-null patients showed a trend towards an increased incidence of SOS (26% *vs.* 15%, *p* = 0.07), diagnosed according to the modified Seattle criteria [51]. In a cohort of 114 Asian Indian pediatric patients undergoing bone marrow transplantation because of β-thalassemia major, a significant increased incidence of SOS was observed in *GSTM1*-null patients compared to those carrying at least one gene copy (46.5% *vs.* 18.3%; *p* = 0.001), as well as a significantly higher BU CL and lower steady-state concentration after the first dose (0.40 ± 0.06 *vs.* 0.33 ± 0.071 L/h/kg, *p* = 0.00001, and 544 ± 184 *vs.* 667 ± 256 ng/mL, *p* = 0.001, respectively) [52]. Based on the previous observation that *GSTM1*-null β-thalassemia major patients showed a 2–4-fold increase in GST-A1 protein and mRNA levels, these authors supposed that the lack of GST-M1 could result in a compensatory GST-A1 effect to explain the pharmacokinetic data: they hypothesized that an increased production of BU metabolites, rather than BU itself, could trigger the hepatocyte damage, either directly or indirectly through depletion of the cellular reduced glutathione (GSH) pool. The authors did not exclude other possible explanations, such as that GST-M1 could play a protective role in sinusoidal cells and that its absence in individuals with the null genotype could be detrimental to cell survival under stress [52]. Indeed, GSTs are known regulators of cellular stress response, apoptosis and proliferation. This function is achieved independently from the well-known GSH conjugating activity and occurs through their ligand-binding capacity; for example, by sequestering members of the mitogen-activated protein kinase family through protein–protein interaction, GSTs negatively regulate the downstream signaling pathway. Similarly, GSTs might interact with other endogenous mediators, such as the 15-deoxy-Δ12,14-prostaglandin J2 (15d-PGJ2) and nitric oxide in dinitrosyl iron complexes involved in hepatocyte proliferation and apoptosis [58].

Bonifazi *et al.* deeply investigated the variants affecting hepatic GSH balance in a cohort of 185 adult patients who underwent allogeneic HSCT from 2005–2009, after a BU-based preparative regimen [59]. They studied 40 polymorphisms at 27 loci, finding that it was a non-synonymous SNP in the GST-A2 isoform (introducing a serine to threonine amino acid substitution at codon 112, S112T) to affect HSCT outcome and BU pharmacokinetics. *GSTA2* S112T serine allele homozygosity was an independent prognostic factor for poorer survival (relative risk (RR) = 2.388) and for increased any time and 100-day TRM (RR = 4.912 and RR = 5.185, respectively). The Ser/Ser genotype also predicts a wider BU area under the concentration-time curve (1214.36 ± 570.06 *vs.* 838.10 ± 282.40 mmol·min) and higher post-transplant bilirubin serum levels (3.280 ± 0.422 *vs.* 1.874 + 0.197 mg/dL). SOS was not included among the clinical phenotype of the study. *GSTA1* rs3957356, rs4715332 and rs1051775 were also investigated with no significant results, although a weak association between TRM and these polymorphisms was observed. Interestingly, the *GSTA2* Ser allele is in strong linkage disequilibrium with the GSTA1*B haplotype.

After the first conjugation of BU with GSH, the formed BU sulfonium ion metabolite is further converted to tetrahydrothiophene (THT). THT is oxidized to the water-soluble sulfolane (Su), and then to 3-hydroxysulfolane, by CYP and flavin-containing monooxygenases (FMOs). Ansari’s group investigated the relationship between common functional alleles in *CYP2C9* (*2 and *3), *CYP2C19* (*2 and *17), *CYP2B6* (*5 and *9), *FMO* (rs2266780, rs2266782 and rs1736557) and the BU/Su metabolic ratio in 44 children receiving a myeloablative regimen with BU (2 h-infusion every 6 h for four days before HSCT), mostly in combination with CPM [48]. The first BU dose was age dependent; pharmacokinetic parameters derived after the first infusion were used to adjust the dosage at the fifth administration to achieve the target steady-state concentration of 600–900 ng/mL; BU and Su plasma levels were assessed after infusion of the ninth BU dose. A higher BU/Su ratio was observed in CYP2C9*2 and *3 allele carriers (mean ± SD: 7.8 ± 3.6 in carriers *vs.* 4.4 ± 2.2 in non-carriers; *p* = 0.003), coherently with the functional impact of these variants in decreasing the enzymatic activity of CYP2C9. An increased incidence of graft failure was observed among patients with a BU/Su >5 compared to those with values <5 (20% *vs*. 0%; *p* = 0.02). In contrast, a significantly higher event-free survival (*i.e*., without relapse and rejection) was observed in patients with malignant disease who carried *CYP2B6* alleles with reduced function on both chromosomes compared to carriers of at least one normal allele (100% *vs*. 40%; *p* = 0.0001). Higher incidence of SOS was observed among patients carrying the normal *CYP2C* allele, although no significant association was detected.

The effect of genetic markers on interpatient BU CL variability has been studied in 65 adult HSCT recipients with the DMET™ array (Affymetrix UK Ltd., High Wycombe, UK), in a high-throughput approach, including the analysis of 1936 SNPs in 225 genes involved in drug metabolism and transport. The seven top genetic markers in six genes (*GSTA5*, *CYP2C19*, *CYP39A1*, *ABCB4*, *SLC22A4* and *SLC7A8*) that emerged as significantly associated with drug pharmacokinetics were selected and validated in a second independent cohort of 78 adult patients [60] and, later on, in a pediatric population of 84 children [61]. Only *GSTA5* SNPs (rs4715354 and rs7746993) remained significantly associated with BU CL in the adult validation cohort. This result was not considered important *per se*, since GST-A5 protein is absent in human tissue [62]; however, the SNP rs4715354 is in linkage disequilibrium with the *GSTA1* SNP rs3957357, −69C>T, missing in the DMET array. *GSTA1* genotype (rs3957357) was subsequently analyzed in the pediatric cohort instead of *GSTA5*. In children, also *CYP39A1* rs2277119 was associated with BU CL. When combined, the *GSTA1* and *CYP39A1* haplotypes explained 17% of the variability in BU CL. CYP39A1 is involved in the conversion of cholesterol to bile acids, and SNP rs2277119 has been associated with a reduced enzymatic function and is associated with increased levels of its substrate, 24S-hydroxycholesterol [63], thus representing a putative risk factor for hepatic toxicity. Furthermore, in younger children (<2 years of age), a stronger effect of *GSTA1* on BU CL was observed. The authors hypothesized that maturation effects account for this difference, and when there is abundant GST activity, which is the case at the age of 2–4 years, a less functional allele in *GSTA1* gene would have a limited effect on BU pharmacokinetics [61].

All of these studies on BU pharmacokinetics revealed the interest of clinicians in optimizing the drug administration schedule on an individual basis. Attention should be focused mainly on the variants GSTA1*B and the deletion of *GSTM1*, as the most promising predictive genetic markers. However, more studies in larger and better characterized cohorts are needed before drawing any firm conclusions.

#### 3.1.2. Other Drugs

Besides BU, several other cancer chemotherapeutic agents are recognized as causes of SOS, including the nitrogen mustard alkylating agent CPM, the platinum coordination complex oxaliplatin and the antimetabolite 6-thioguanine (6-TG). These drugs are commonly used in pre-transplant therapeutic protocols for malignancies, and variants in pharmacogenes known to interfere with the metabolism of these drugs have been investigated for their influence on the occurrence of sinusoidal toxicities, in both HSCT and no HSCT clinical settings.

CPM is a prodrug whose active metabolites, phosphoramide mustard and acrolein, are derived from a multistep activation pathway that begins with the hepatic oxidation of CPM to 4-hydroxy-CPM mediated by the CYP isoforms 3A4, 2C9 and 2B6. Samples of 107 leukemia adult and pediatric patients of European origin given HLA-identical HSCT were genotyped for candidate polymorphisms, among which are the non-synonymous SNPs *2 (C64T, Arg22Cys), *3 (C777A, Ser259Arg), *4 (A785G, Lys262Arg), *5 (C1459T, Arg487Cys) and *6 (G516T, Gln172His) in the *CYP2B6* gene. In 68% of cases, conditioning therapy consisted of a combination of BU, CPM and, eventually, other drugs; SOS was classified according to Seattle criteria, and the cumulative incidence at Day +100 was 14% (95% CI 7.4–20.6). In univariate analysis, CYP2B6*6 was associated with higher incidence of SOS if both donor and recipient were wild-type (GG); the multivariate analysis confirmed the significant role of donor genetic contribution: 20.6% (10.6–30.7) of patients transplanted from CYP2B6*6 GG carriers developed mild to severe SOS *vs.* 4.5% (0.0–10.8) of those transplanted from GT/TT subjects (HR = 3.49; 95% CI = 1.12; 10.88; *p* = 0.03) [47]. The CYP2B6*6 variant shows a dual functional impact: the mutant allele leads to a decreased expression level, although the resulting variant protein has an enhanced catalytic ability [64]; individuals with wild-type genotypes are thus supposed to have higher enzymatic activity and to be better producers of CPM active metabolites. Results of retrospective analysis, such the above mentioned ones, need to be confirmed by integrating genetic data with CPM drug monitoring in other patient cohorts.

Even the antibiotic vancomycin or the antifungal amphotericin B, as well as antiviral prophylactic treatment with acyclovir represent other risk factors for developing SOS [38,40]. Among other drugs, antifungals, such as itraconazole, can increase busulfan exposure, probably because of inhibition of busulfan metabolism in the liver, and, therefore, may affect incidence of SOS [65]. So far, these interactions have not been systematically considered, and future prospective studies on this aspect are needed.

#### 3.1.3. Defibrotide

Defibrotide is a mixture of polydeoxyribonucleotide of mammalian origin, which was initially identified as an adenosine receptor agonist [66]. In the current HSCT setting, this pharmacological agent has been investigated for SOS prophylaxis [67], but evidence of its safety with respect to hemorrhagic complications support its role also in SOS treatment. Indeed, defibrotide has relatively low risk of systemic bleeding, in contrast to other systemic anticoagulants or thrombolytics used in this regard. Defibrotide does not have a direct anticoagulant function, but shows pleiotropic antithrombotic properties acting on the fibrinolytic pathway: it enhances the expression of tissue plasminogen activator (tPA), a serine protease that catalyzes the conversion of plasminogen to plasmin; it enhances the activity of plasmin itself and reduces the level of plasminogen activator inhibitor type-1 (PAI-1) [68]. Interestingly, plasma levels of PAI-1 have been shown to significantly increase 48 h after starting the first BU infusion in 18 children preconditioned for HSCT, and genome-wide transcriptional profiling of BU-treated and -untreated cells revealed a differential expression of genes related to the coagulation system modulated by TGF-β1, rather than TNF-α [69]. These results give rationale to the clinical practice of combining defibrotide in a BU-based conditioning regimen. Moreover, among other potential mechanisms of action, defibrotide regulates also the levels of important vascular mediators, e.g., reducing the endothelial cell surface procoagulant tissue factor and stimulating the release of vasodilators nitric oxide (NO) or prostacyclin.

Pharmacogenetic studies on defibrotide are actually missing; however, it is reasonable to suppose that the individual response to defibrotide could be influenced by genetic variants affecting the coagulation pathway (see below). In this regard, *PAI-1* 4G/5G Ins/Del polymorphism at position −675 (rs1799889) is the ideal candidate: the 5G allele affects *PAI-1* gene transcription lowering the plasma PAI-1 levels thus inducing a potential hypofibrinolytic state.

### 3.2. Variants Affecting SOS Susceptibility

Polymorphisms in genes related to different functional pathways (e.g., cytokines and their receptors, genes involved in the synthesis and metabolism of cellular antioxidants, clotting and fibrinolytic factors) have been taken into account to understand the genetic basis of SOS susceptibility in HSCT settings.

Interleukin (IL)-1β is a pro-inflammatory cytokine secreted by both immune and non-immune epithelial tissues early in the inflammatory response. A total of 76 pediatric patient/donor pairs transplanted between September 2000 and December 2004 at St. Jude Children’s Hospital (Memphis, TN, USA) were genotyped for interleukin IL-1β polymorphism rs16944, a C>T substitution at position 511 that results in the loss of a putative AP-2 binding site and in higher secretion of IL-1β protein [54]. Interestingly, donor, but not patient, TT genotype was associated with higher cumulative incidence of Grades III–IV hepatic SOS at three months after transplantation, as defined according to the National Cancer Institute Common Toxicity Criteria, Versions 2 and 3 (25% ± 13.1% in TT, 2.9% ± 2.9% in CT and 3.6% ± 3.6% in CC; *p* = 0.024). The authors suggested that local release of IL-1β originating from damaged host tissues could be further propagated by allo-stimulated donor leukocytes, in particular by higher IL-1β producers. Furthermore, the cytokine prompts the release of other pro-inflammatory cytokines, acts as a procoagulant and upregulates the bystander endothelial cell expression of the adhesion molecules, such E-selectin, vascular cell adhesion molecule-1 (VCAM-1/CD106) and intercellular cell adhesion molecule-1 (ICAM-1/CD54).

Pretransplantation serum hyperferritinemia is considered a risk factor for SOS in HSCT patients [70,71,72]. Even in pediatric patients, hyperferritinemia is routinely assessed, and high levels are usually treated with iron chelating agents to reduce liver toxicity. Ferritin is the ubiquitous cytosolic protein that stores iron inside the cells; its circulating isoform is used as a clinical marker for total body iron, because it correlates with the directly measured content in the liver, where the excess of iron is stored [73]. Being a potent catalyst for free-radical reactions that generate highly reactive oxygen species able to irreversibly damage cellular constituents, exceeding iron is highly toxic for its propensity to induce oxidative stress. One hundred sixty six HSCT adult recipients transplanted between 1995 and 1999 were prospectively evaluated for SOS (Baltimore criteria) and for candidate polymorphisms in the hemochromatosis (*HFE*) and carbamoyl-phosphate synthetase (*CPS*) genes, affecting the iron metabolism. The HFE protein regulates the iron cellular absorption [74,75]. Individuals that carry at least one variant allele for the major non-synonymous polymorphism rs1800562 in the *HFE* gene (845 G>A in exon 4, in which a tyrosine is substituted for a cysteine at position 282) have elevated plasma levels of reactive iron; carriers of the mutated genotype developed one hereditary form of hemochromatosis, an iron overload disorder. NO acts as a local antioxidant, vasodilator and platelet antagonist and regulates cellular iron metabolism during stress and inflammation. CPS catalyzes the rate-limiting step of the hepatic urea cycle required for the synthesis of NO precursors, arginine and citrulline. SNP rs1047891 in the *CPS* gene (a C>A mutation in exon 36 that changes threonine to asparaginase at position 1405) leads to significantly higher plasma levels of NO and, thus, to an increased protection from oxidative stress. Risk of SOS was significantly higher in carriers of at least one 282Tyr allele (RR = 3.7, 95% CI 1.2–12.1) and increased progressively with 282Tyr allelic dose (RR = 1.7, 95% CI 0.4–6.8 in heterozygotes; RR = 8.6, 95% CI 1.5–48.5 in homozygotes). The *CPS* A allele, which encodes a more efficient urea cycle enzyme, counteracted the risk of SOS associated with *HFE* 282Tyr [46].

In a very recent report, Seifert *et al.* investigated the role of two genetic variants (rs4693608, G>A, and rs4364254, C>T) in the heparanase (*HPSE*) gene [53]. *HPSE* encodes for an endo-β-d-glucuronidase that degrades the saccharide chains of heparan sulfate proteoglycans, physiological anticoagulant located in the endothelial layer of blood vessels. Increased HSPE function has been associated with prothrombotic states, angiogenesis and metastatic processes; the rs4693608 SNP was found to influence the *HPSE* gene expression in activated mononuclear cells from healthy volunteers of different ethnic origins: possessors of the AA genotype exhibited upregulation of *HPSE*, whereas individuals with the GG genotype showed downregulation or no effect [76]. Seifert *et al.* retrospectively analyzed 160 pediatric and adult patients who underwent allogeneic HSCT between January 2000 and December 2011 after a myeloablative conditioning regimen. The majority of patients (88.8%) received SOS prophylaxis with defibrotide. The authors investigated the rs4693608 and rs4364254 role in early- and late-onset SOS (before and after Day +20, respectively). Patients carrying at least one rs4693608 G allele had a significantly reduced incidence of SOS on Day +100 after HSCT compared to patients with genotype AA (4.7% *vs.* 14.3%, *p* = 0.038); SOS in patients carrying the rs4364254 C allele was significantly decreased in comparison to patients with the TT genotype (2.3% *vs.* 14.7%, *p* = 0.004), and interestingly, no patient with the CC genotype developed SOS. In multivariate analyses, *HPSE* polymorphisms turned out to be significant independent risk factors (*p* = 0.030) for the development of SOS. Subset analysis considering combined genotypes revealed that patients with mutations in both SNPs (AA-TT) had the highest SOS incidence (16.7%), in contrast to patients to a favorable wild-type GG-CC combination who did not develop SOS (0%); residual genetic combinations grouped together were in-between (4.9%). The difference among the three groups was significantly different (*p* = 0.035), suggesting the need of further analyzing these SNPs in other clinical trials [53].

Risk factors associated with thrombophilia could also favor SOS development. Eighty-nine HSCT adult and adolescent patients transplanted between 2000 and 2002 in one singe German center were genotyped for inherited genetic variants known to predispose to thrombosis (Factor V Leiden *F5* gene rs6025 (G1691A), prothrombin *F2* rs1799963 (G20210A), *MTHFR* rs1801133 (C677T), *PAI-1* rs1799889, glycoprotein IIIa (*GPIIIA*) PI a1/a2 polymorphism, fibrinogen-β-chain (*FGB*) rs1800790 and the angiotensin-converting enzyme (*ACE*) intron 16 I/D polymorphism) and were prospectively evaluated for hemostatic complications, including bleeding and thrombotic events. Patients were mainly conditioned by reduced intensity conditioning regimen (70.8%) and without BU (~90%), received intravenous heparin (100 IU/h) prophylactically during the whole transplantation period and were prospectively evaluated for hemostatic complications, including bleeding and thrombotic events [45]. An increased frequency of the 4G allele in the *PAI-1* gene was observed in patients with catheter thromboses (85.7% *vs.* 55.1%; *p* = 0.022, relative risk = 5.7), as well as in patients with hepatic SOS. However, no significant result could be designed for these patients, because only three cases of SOS confirmed by transjugular liver biopsy were registered. Thrombophilic polymorphisms rs6025 (G1691A) in *F5* and rs1799963 (G20210A) in *F2* were retrospectively characterized in 209 adult and pediatric allogeneic HSCT patients transplanted between 1984 and 1996 and 10 consecutive enrolled HSCT children in 1997 [49,50]. In the former study, 15 out 22 cases of SOS, histologically and clinical assessed, were genotyped. Two of these patients were heterozygous for *F2* rs1799963, whereas none had the *F5* variant allele. By comparison to respective control groups, the authors suggested rs1799963 as a risk factor of SOS [49]. In contrast, Akar *et al.* analyzed a small group of 10 patients: three out of four patients developed SOS had *F5* heterozygosity, whereas none had the *F2* mutation, suggesting the importance of the rs6025 polymorphism [50]. In an Italian study on 69 adult HSCT, thrombotic complications occurred in 4.3% of cases, but only one patient developed SOS; thus, the impact of these variants could not be assessed [77]. In conclusion, the role of inherited prothrombotic genetic risk factors in the onset of SOS in HSCT patients still needs to be investigated, as no conclusive result emerged because of the smaller cohorts analyzed; larger prospective studies are hence required.

## 4. Variants Affecting Transplant-Related Mortality

Deaths occurring after HSCT and unrelated to the primary disease, *i.e.*, occurring without progression or relapse of the underlying illness, are commonly referred to as TRM or as “non-relapse mortality”, as recently suggested by EBMT. Improved HSCT clinical practices, with changes in stem cell sources, different GVHD prophylaxis and better management of infections, have generally decreased TRM over the past few decades, despite the increasing numbers of unrelated donors and of transplanted patients at advanced stage disease [78,79,80,81,82]. In the AIEOP-HSCT context, the incidence of TRM within the first 100 days post-transplant has actually achieved 10%–15%, as emerged from the recent analysis on 3730 consecutive allogenic HSCT carried out in 26 Italian centers between 1999 and 2012 [82], and in the 1296 transplanted patients in the previous 14-year period (from January 1985–December 1998) [83]. In a pediatric population of 636 consecutive ALL patients who underwent HSCT in 13 AIEOP transplant centers between January 1990 and December 1997, the toxicity of any organ, but mucosa and gut, was positively correlated with early death; moderate and severe toxicity to heart, lungs, liver and kidneys significantly increased early TRM, with estimated relative risks of 9.1, 5.5, 2.7 and 2.8, respectively, as compared to absent or mild toxicity [14].

TRM are more likely related to toxicities arising from patient’s pre-transplant treatments. Previously given chemotherapies, irradiation and/or myeloablative conditioning regimens, as well as calcineurin inhibitors commonly included in pre-transplant regimen for GVHD prophylaxis inevitably damage patient mucosal and vascular endothelial cells [84]. Vascular stress occurred also after HSCT, during the transmigration of allo-specific lymphocytes across the endothelial cells: as a consequence of the enhanced expression of antigen-presenting and adhesion molecules on their surface, the endothelium is transformed in a pro-coagulant surface, thus predisposing to systemic coagulation disorders [85]. Indeed, hemostatic changes and thrombotic events are frequent in HSC transplanted patients, and severe manifestations of early coagulopathy (particularly those leading to organ dysfunction, such as SOS) might be life-threatening [86]. Thyagarajan *et al.* investigated the genetic variants of vascular endothelial adhesion molecules in 425 allogeneic HSCT recipient-donor pairs (27.3% of recipients under 20 years of age) and their association with GVHD (acute and chronic) and TRM occurring within one year [87]. Four nonsynonymous SNPs were considered: rs5498 (A>G in exon 6, K469E) in the gene encoding for the intracellular adhesion molecule 1 (*ICAM1*); rs668 (L98V) and rs1131012 (R643G) in platelet/endothelial cell adhesion molecule 1 (*PECAM1*); rs2229569 (P213S) in L-selectin (*SELL*). After adjustment for clinical covariates, significantly decreased risk of TRM was reported only for rs5498, in both recipients (HR = 0.67, 95% CI = 0.50–0.89, *p* = 0.01) and donors (HR = 0.73, 95% CI = 0.55–0.95, *p* = 0.02). In recipients, significance was also maintained after adjustment for aGVHD as a time-dependent covariate, suggesting an influence of the polymorphism on TRM independent of aGVHD. Non-identity, defined by the authors as “genotype mismatch within each recipient-donor pair” and non-compatibility, defined as “recipient allele that could be recognized as foreign by the donor” were not associated with TRM. Thus, the authors suggest that none of these SNPs serve as a miHA [87]. The G minor allele of rs5498 (or of SNPs in linkage disequilibrium) was significantly associated with higher soluble ICAM-1 levels in previous studies [88,89,90,91,92]. ICAM-1 participates in inflammatory processes by promoting adhesion of leukocytes to vascular wall endothelium.

In a mouse model, animals with P- and E-selectins on the surface of vascular endothelial cells showed markedly reduced incidence of SOS, demonstrating a major role for blood leukocytes [93]; however, no clinical pharmacogenetic investigation on genes encoding these selectins and HSCT has been performed so far.

Thrombomodulin (TBHD) is a transmembrane glycoprotein expressed on endothelial cells and acts as an important negative regulator of the coagulation process. When THBD interacts with thrombin, the latter switches its substrate specificity from fibrinogen to protein C, which is in turn activated and interacts with protein S. The protein C–protein S complex is able to dampen coagulation by inactivating the activated pro-coagulation factors Va and VIIIa and to reduce the inflammatory process by inhibiting the production of inflammatory cytokines and the transduction of nuclear-factor-κB (NF-κB) signaling in monocytes [94]. A recent study published in the Journal of Clinical Oncology evaluated the association between seven SNPs of the endothelial *THBD* gene and TRM in two cohorts of HSCT patients, resulting in three non-overlapping high risk genotypes, *i.e*., CC for rs1962, TT for rs1042579 (A473V) and GG for rs1042580. Subjects carrying one of these genotypes had more than a two-fold risk of non-relapse mortality in comparison to the others (HR = 2.31, 95% CI = 1.36–3.95, *p =* 0.002 in the training cohort of 306 patients; HR = 2.18, *p* = 0.006 in the validation and 321 patients), an association that remained significant also in multivariate analyses after adjustment for clinical variables [95]. More specifically, the *THBD* SNPs were TRM predictive only in patients who developed GVHD (HR = 3.03; 95% CI = 1.61–5.68; *p* < 0.001), but not in those who did not, although *THBD* SNPs was not associated with the incidence of acute GVHD [95].

## 5. Controversial Issues in HSCT Studies

The first consideration emerging from this report is that no single polymorphism results in being unequivocally clinically relevant in the plethora of variants considered. Although some of them seem to be more interesting than others (e.g., *GSTA1* and *GSTM1* variants affecting BU pharmacokinetics and SOS), they have not received sufficient evidence to justify their predictive use in HSCT practice so far. Most of the pharmacogenetic results are controversial; however, it should be noted that they refer to different cohorts in terms of age, gender, ethnicity, patient underlying diseases and previous pharmacological approaches, donor source, HSCT myeloablative conditioning and prophylactic treatments and that even within the same study, the analyzed population is not homogenous. This raises the possibility that low penetrance genetic contribution on HSCT outcome is hidden and that the lack of coherence across results reflects the confounding heterogeneity in patient characteristics. Moreover, HSCT outcome and post-transplant courses are multifactorial events, and it is unlikely that a single polymorphism could emerge as the unique determinant of such complex phenotypes. It is more likely that several genetic variants affecting the same biological pathway impact on easy-measurable drug pharmacokinetic parameters and, thus, indirectly on outcome. It would be particularly important to analyze separately at least pediatric and adult HSCT cohorts: age influences both the individual metabolic profile, as a consequence of different hormonal stimulation or physio-pathological conditions occurring during life, and dosage and regimens of the therapeutic protocols that are administered. Race also introduces great discrepancies among investigated populations, since allelic frequencies could vary greatly according to genetic ancestry, so that studies regarding a population of different origin should not be compared. Surely, analysis should be stratified for diseases and previous treatments. HSCT clinical phenotypes are also not uniformly assessed across studies, particularly when univocal guidelines are missing at the national or international level. For example, the severity of SOS episodes could be graded according to different criteria (e.g., modified Seattle or Baltimore criteria, histological determination).

The second consideration is that studies are often performed without an adequate number of patients and that further investigations with a larger sample size are required to support genetic findings with greater statistical confidence before any statement could be made. Cohorts are generally very small, and if not, they include patients who underwent different HSCT clinical approaches because of being enrolled over many years, thus introducing the already mentioned confounding heterogeneity in treatment protocols. Multicentric studies overcome these limitations. However, they pose the problem of accuracy, quality and standardization in samples and clinical data collection among the medical units involved. In this context, long-term experience and guidelines developed by national and international networks (e.g., AIEOP and EBMT, respectively) are fundamental. In Italy, AIEOP is also organized to create a common DNA BioBank and a centralized database of clinical data, as reported in previous pharmacogenetic studies on ALL patients [96,97].

Finally, correlation between donor and patient genotypes and patients drug pharmacokinetics is a key issue in HSCT studies. The third consideration that can be drawn is that prospective studies combining pharmacogenetic information with pharmacokinetic analysis and drug monitoring should be preferred to retrospective approaches.

## 6. Conclusions

Allogeneic HSCT is a complex medical procedure, particularly demanding for patients affected by severe hematological and autoimmune illnesses who have already received aggressive and prolonged drug treatments without success. HSCT outcome depends on several variables, both patient- and transplant-related, whose knowledge has certainly improved clinical practices over years; however, a large inter-patient variability in post-transplant course is still observed, and recovery and complications are only partially predictable by routine clinical monitoring. Recipient and/or donor genetic background, as well as the genetic discrepancies in the pair could provide insight into the individual response to pharmacological agents. This article reviews the current literature on the role of common polymorphisms in allogeneic HSCT outcome, with particular attention to the pharmacogenetic variants able to influence drug disposition, metabolism and the mechanisms of action directly. The identification of genetic markers able to predict the risk of developing severe HSCT complications would be extremely useful for better tailoring pharmacological myeloablative and immunosuppressive treatments to HSCT patients and improving their outcome. However, large multicentric, highly standardized prospective studies are needed to identify potential pharmacogenetic markers with sufficiently strong evidence to be used in clinical practice.
